# The Role of Fructose as a Cardiovascular Risk Factor: An Update

**DOI:** 10.3390/metabo12010067

**Published:** 2022-01-12

**Authors:** Stefan-Sebastian Busnatu, Teodor Salmen, Maria-Alexandra Pana, Manfredi Rizzo, Tiziana Stallone, Nikolaos Papanas, Djordje Popovic, Denisa Tanasescu, Dragos Serban, Anca Pantea Stoian

**Affiliations:** 1 Cardiology Department Bucharest, Carol Davila University of Medicine and Pharmacy, 050474 Bucharest, Romania; stefan.busnatu@umfcd.ro (S.-S.B.); maria.alexandra.pana@drd.umfcd.ro (M.-A.P.); 2 Department of Diabetes, Nutrition and Metabolic Diseases, “Prof. Dr. N.C.Paulescu” National Institute of Diabetes, Nutrition and Metabolic Diseases, 030167 Bucharest, Romania; teodor.salmen@drdumfcd.ro; 3 Department of Health Promotion Sciences Maternal and Infantile Care, Internal Medicine and Medical Specialties (PROMISE), School of Medicine, University of Palermo, 90100 Palermo, Italy; manfredi.rizzo@unipa.it; 4 Italian Council and Pension Funds for Biologist Enpab, 00153 Rome, Italy; presidenza@enpab.it; 5 Second Department of Internal Medicine, Diabetes Centre-Diabetic Foot Clinic, Democritus University of Thrace, University Hospital of Alexandroupolis, 68100 Alexandroupoli, Greece; papanasnikos@yahoo.gr; 6 Medical Faculty, University of Novi Sad, 21102 Novi Sad, Serbia; pitstop021@gmail.com; 7 Fourth Department of Dental Medicine and Nursing, Faculty of Medicine, ‘Lucian Blaga’ University, 550024 Sibiu, Romania; denisa.tanasescu@ulbs.ro; 8 Department of General Surgery, Carol Davila University of Medicine, 020021 Bucharest, Romania; 9 Forth Department of General Surgery, Emergency University Hospital Bucharest, 050098 Bucharest, Romania; 10 Department of Diabetes, Nutrition, and Metabolic Diseases, Carol Davila University of Medicine, 050474 Bucharest, Romania; ancastoian@yahoo.com

**Keywords:** fructose, cardiovascular disease, nutrition, risk factors, lipoproteins

## Abstract

There is increasing presence of fructose in food and drinks, and some evidence suggests that its higher consumption increases cardiovascular risk, although the mechanisms still remain not fully elucidated. Cardiovascular diseases (CVD) are still responsible for one-third of deaths worldwide, and therefore, their prevention should be assessed and managed comprehensively and not by the evaluation of individual risk factor components. Lifestyle risk factors for CVD include low degree of physical activity, high body mass index, alcohol consumption, smoking, and nutritional factors. Indeed, nutritional risk factors for CVD include unhealthy dietary behaviors, such as high intake of refined foods, unhealthy fats, added sugars, and sodium and a low intake of fruits, vegetables, whole grains, fiber, fish, and nuts. Even though there is no definitive association between CVD incidence and high consumption of total sugar, such as sucrose and fructose, there is, however, evidence that total sugars, added sugars, and fructose are harmfully associated with CVD mortality. Since high fructose intake is associated with elevated plasma triglyceride levels, as well as insulin resistance, diabetes hyperuricemia, and non-alcoholic fatty liver disease, further longitudinal studies should be conducted to fully elucidate the potential association between certain sugars and CVD.

## 1. Introduction: The Global Problem of CVD and the Nutrition Role of Sugars and Fructose

Cardiovascular disease (CVD) represents a group of disorders of the heart and/or blood vessels that includes peripheral arterial disease, stroke, coronary heart failure, high blood pressure, and other vascular and/or cardiac conditions, which are the leading cause of morbi-mortality worldwide and represent a significant burden for healthcare systems [1,2]. For example, a valuable tool to achieve the atherosclerosis extent is the carotid intimal-medial technique, which is reported to be predicted by risk factors from an early age of 8 to 11 years [3].

Prevention and evolution of CVD are reported to be linked with diet and nutrition, respectively, by various versatile aspects, such as nutrients, food, or eating patterns, each modified over time in connection with the recent discoveries. Anyhow, risk factors of CVD development are modifiable, including diet, smoking, lifestyle, or environment, while others are unmodifiable, such as genetic factors, age, gender, or history [4]. Prevention of CVD, its severe consequences, and the proinflammatory effect of the Western type of diet, respectively, as well as frequent consumption of processed and fast food and a diet with high intake of red-meat-based protein, sugar, fat, cholesterol, and salt, is promoted by international guidelines [2,5].

In addition, such guidelines recommend positive lifestyle modifications, including cessation of smoking, engaging in regular physical activity, and attention to the maintenance of proper weight, alongside positive nutritional practices, such as a healthier intake of fruits, vegetables, fish, healthy oils, nonfat dairy, seafood, and whole-grain nuts, such as included in a Mediterranean diet [2,5]. Dietary regimens include composition, calorie intake, and feeding patterns; and, alone or combined with the lifestyle factors that influence CVD, modulation of low-grade chronic inflammation [2]. Obesity and diabetes mellitus (DM) are both proinflammatory state conditions that, alongside a high intake of glucose and macronutrients and by chronic overnutrition, lead to an increase in insulin resistance and interference with insulin-signaling mechanisms, thus decreasing its anti-inflammatory effect [6].

## 2. Fructose and Nutrition: Sources of Fructose and Its Mechanism of Action

Fructose, as compared to glucose, enters more slowly into the bloodstream, developing lower levels but with a higher persistence. The general intake in a Western diet is of 49 g fructose/day, only 8 g being from natural sources [7]. The metabolization process mainly occurs in the liver, where it is converted to trioses and released as lactate into the bloodstream or converted to glucose via gluconeogenesis, which is removed or stored as glycogen. Because of the high gradient of blood entering and leaving the liver, the pancreas, alongside the liver, is exposed to high fructose concentrations. Teff K et al. reported that high fructose intake determines a lower insulin response of 65%, glycemic excursions of 66%, and GLP-1 secretion of 112.7 pg/mL, as compared to 46 pg/mL of glucose intake [8]. Furthermore, it is released from the liver as glucose, fatty acids, or lactate and oxidated in extrahepatic tissues or increases the exogenous carbohydrate rate of oxidation [7]. The chemical formula of fructose is C_6_H_12_O_6_ [9].

The sources of fructose from the daily human diet are primary-caloric sweeteners (sucrose, honey, apple juice, etc.) and secondary (simple sugars from nuts, fruits, or vegetables) [10]. The recent data report that saturated fats lose the position of the main culprits of CVD, in favor of a high intake of carbohydrates, alongside food with high-glycemic-index or glycemic-load diets. In a study of adult rats, De Gastro U.G. et al. reported that even though both a high-fat diet and a high-fructose diet increased heart rate and mean blood pressure, the former also increased the visceral lipid store from 7.24 g to 8.13 g, favoring metabolic syndrome, while the latter favored the increment of seric lipids (triglycerides from 0.34 mmol/L to 1.02 mmol/L; total cholesterol from 2.26 mmol/L to 2.97 mmol/L; and HDL-cholesterol from 0.38 mmol/L to 1.8 mmol/L), along with their accumulation in the liver and kidney [11]. The link between glycemic index/glycemic load and CHD is influenced by gender—higher in females, possibly because of a greater decrease in HDL and a greater increase in triacylglycerol in response to high-glycemic diets; and by the body mass index (BMI)—overweight and obese patients, even after adjusting for age, smoking status, amount of physical activity, alcohol consumption, and total energy intake, possibly because of preexisting levels of adiposity; and by the higher demand for insulin that exacerbates insulin resistance and related lipid metabolic disorders [2]. On the other hand, Lakhan S.E. et al. reported that the lower level of satiety from fructose intake compared to glucose or sucrose leads to body weight gain and promotes obesity [12]. Pereira R.M. et al. report not only on the increment of fructose consumption, but its harmful effects, such as favoring installation of chronic and subclinical inflammation, and propose that regular aerobic physical activity, strength training, or a combination of both may reverse these parameters, limiting the obesogenic character of fructose intake [13].

Bandini L. et al. reported that obese adolescents do not eat more high-calorie, low-nutrient-dense foods, such as chips and soda, as compared to non-obese adolescents and, what is more, the latter had a greater caloric intake from candy, baked goods, and ice cream [14]. Fructose and glucose are found to be an increasing trend in sugar-sweetened beverages, and their intake is also increasing. Still, the literature data report that their consumption is not compensated by a reduction in the caloric intake of other foods, leading to a positive balance in the caloric intake, as well as to increased body weight, levels of plasma triglycerides, visceral adipose tissue, and muscle fat and liver fat, mainly due to fructose intake, rather than to glucose in sugar [15].

The liver uptake of dietary glucose is estimated to be almost 20%, while dietary fructose is majoritarian. Secondly, only 50% of it is metabolized to glucose, leading to a dietary conversion of glucose-to-fructose ratio in the liver of 3:1 [9]. It is important to emphasize that extreme study protocols for fructose overfeeding lead to abnormal metabolic shifts, including glucose-to-fructose ratios, which is highly risky for the individuals because, physiologically, humans consume both fructose and glucose [9].

## 3. Fructose and Cardiovascular Risk

An individual’s susceptibility to developing a CV event within a predefined period of time establishes his CV risk factor. In recent years, much emphasis has been placed on lifestyle for prevention of CVD. Consequently, international cardiology guidelines place lifestyle as the first line in CVD prevention. In medical terms, a healthy lifestyle corresponds to a physically active life, maintaining an average body mass index, following a balanced diet, low alcohol consumption, and abstinence from nicotine products [16]. Regarding fructose-consumption recommendations, the American Heart Association considers that a prudent upper limit of fructose consumption is 50% of the discretionary calorie allowance relative to the daily caloric number required for energy production. The discretionary calorie allowance, which is left over after meeting the nutrient requirements, is equally divided between solid fats and added sugars. Relating on the caloric needs of each person, the amount of sugar added may vary from 5 teaspoons per day (for an average woman with a daily caloric requirement of 1800 calories) to 9 teaspoons per day (for an average adult man with a daily caloric requirement of 2200 calories) [17]. Quan he Yang et al., 2014, observed during an epidemiological study that most adults from the United States of America consume a much larger amount of added sugar than recommended, with a consequent increase in the risk of death from CVD [18].

The evolution of our society has led to an involution in terms of food consumed. We distanced ourselves from nature out of a desire to speed up food production and slow down food degradation. In addition to these aspects, consumer society has encouraged the improvement of food taste, which has been achieved by the addition of sweeteners. One of the most widely used industrial sweeteners is fructose, excessively consumed, especially in Western diets [19]. In 1998, a health status, health behavior, food, and nutrient-consumption surveillance system was initiated in Korea, entitled the Korea National Health and Nutrition Examination Survey (KNHANES). It aims to assess the dietary patterns of the population in order to establish strategies for health-promotion plans and programs. KNHANES uses stratified multistage cluster sampling and the rolling sampling method to ensure population representativeness. With assessments initially conducted every three years, then annually (starting from 2005), they are composed of a health interview, nutrition survey, and health examination. According to the Korea National Health and Nutrition Examination Survey, the consumption of sugar-sweetened beverages and processed foods increased 3.7 times between 1998 and 2014, with a preference in adolescents. Among artificially sweetened drinks, the carbonated beverages were most frequently responsible for the excessive amount of sweetener in the group of children between 6 and 11 years old. This study also found a positive association between excessive sugar and carbonated beverages and an increased incidence of childhood obesity [20]. Therefore, the consumption of artificially sweetened foods, especially among children, should be monitored globally in order to organize effective prevention campaigns concerning childhood obesity. Furthermore, these observations led to the study of fructose adverse effects on both animals and humans to clarify whether there is a link between excessive fructose consumption, obesity, and CVD [21]. In Table 1, we summarize the main findings from a few preclinical and clinical studies evaluating fructose consumption on cardiometabolic parameters.

A recent study published by SooYeon Yoo et al., 2016 [21], aimed to determine whether high dietary fructose increases CV risk in growing rats. Thus, they compared four experimental groups: a regular diet group, a high-fructose diet group, a high-fat diet group, and high-fructose with high-fat diet group for a period of eight weeks. At the end of this term, they compared body weight, total-fat weight, serum glucose, insulin, lipid profiles, proinflammatory cytokines, abdominal aortic wall thickness, and eNOS and ET-1 mRNA expressions. The analysis of these parameters led to important observations. First, although a high-fructose diet does not affect body weight, body composition changes in favor of fat compared to the regular diet-fed group. Second, when exposed to high-fructose food, triglyceride synthesis increases in rats, an aspect responsible for the onset of dyslipidemia and obesity, two significant promoters of CVD. Third, abdominal aortic wall thickness was greater in high-fructose and high-fat diet rats compared to the regular-diet group. Additionally, high-fructose food content leads to endothelial dysfunction in rats, by mRNA increased expression of ET-1, an important vasoconstrictor. The cited study did not show abnormal values in serum glucose, insulin levels, and proinflammatory cytokines. The authors concluded that a 30% fructose diet increases the CV risk factor by body fat growth, dyslipidemia, enhancement of aortic wall thickness, and endothelial dysfunction [21].

Perhaps one of the greatest health dangers is the proverbial beautiful apple on the outside and rotten on the inside. Even in the presence of a normal body mass index, nutritional imbalance can cause damage to the body. In an increasingly outward-looking society, we need to find the proper methods to redirect patients to what matters: health, vitality, and body strength. We consider that it is not enough to be in the normal weight- range if one faces dyslipidemia, a high percentage of body fat, and atherosclerotic lesions of the main arteries. From an endocrinological point of view, the link between increased fructose intake, increased insulin resistance, hyperinsulinemia, and endothelial dysfunction has been demonstrated [22]. Apparently, these mechanisms, alongside a more pronounced action of the sympathetic nervous system, underlie the occurrence of hypertension. Tran L.T., Yuen V.G et al., 2009 [23], evaluated the mechanisms of fructose-induced insulin resistance and hypertension in fructose-fed rats. The vicious circle incriminated in the mechanism of hypertension is based on vasoconstriction. Excess fructose leads to insulin resistance, which activates the sympathetic nervous system.

In consequence, hyperinsulinemia stimulates continuously increased sympathetic nervous-system activity. Although unclear which abnormality generates the next one, it is certain that chemical sympathectomy leads to lower insulin levels and lower blood pressure. The authors conclude that although the activation timing of the central nervous system is uncertain, it definitely represents a central part in hyperinsulinemia and hypertension development in animal models [23].

A review by Te Morenga LA et al., 2014 [24], that included 39 studies aimed to confirm the existence of a link between excessive sugar consumption, increased lipid levels, and arterial hypertension. Thus, diets with a high sugar content, compared to those with a low sugar content, increase triglyceride concentrations, with a mean value of 0.11 mmol/L, total cholesterol, with 0.16 mmol/L, low-density lipoprotein cholesterol, with 0.12 mmol/L, and high-density lipoprotein cholesterol, with 0.02 mmol/L. On the other hand, the effects of sugar intake on blood pressure values were more significant in trials over eight weeks. In those cases, systolic blood pressure values had an increased mean value of 6.9 mmHg, and diastolic blood pressure had a high mean value of 5.6 mmHg. Dietary sugars certainly influence lipid levels and blood pressure levels, regardless of the variation in body mass index [24].

**Figure 1:**
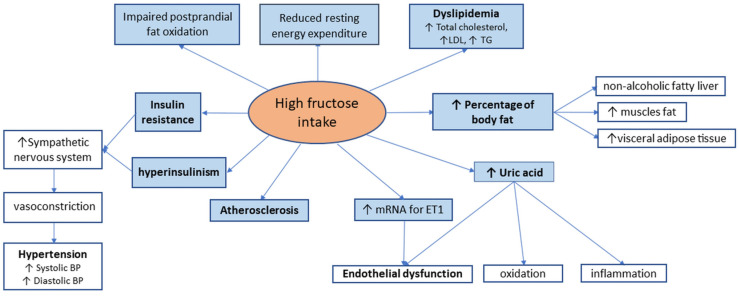
Clinical effects of high fructose consumption.

In humans, the consequences of high fructose consumption have similar effects to those seen in animal models, leading to non-alcoholic fatty liver (NAFLD) disease, dyslipidemia, insulin resistance, reduced resting energy expenditure, and impaired postprandial fat oxidation [25]; this is summarized in Figure 1. Furthermore, fructose is the only carbohydrate that generates uric acid during its metabolization. Uric acid levels rise a few minutes after high-fructose meals, with circulatory levels remaining high, even in the late postprandial phase [26]. This is the consequence of hepatic phosphorylation of large quantities of fructose, which produces adenosine triphosphate depletion, thus stimulating the production of uric acid [27]. Uric acid induces inflammation, oxidation, and endothelial damage, aggravating atherosclerotic lesions in CVD patients. Decreased levels of uric acid through appropriate therapy lead to improvement in metabolic syndrome. This is evidence of the direct involvement of uric acid in developing insulin resistance and metabolic syndrome in patients on high-fructose diets [28].

Regarding potential mechanisms involved in the increased CV risk induced by the overconsumption of fructose, there is evidence that higher fructose intake alters lipoprotein distribution, with an increase in atherogenic small, dense LDL particles; notably, this has been shown over the years in both adults [25,29] and children [30,31]. This has a significant clinical relevance since higher levels of small, dense LDL particles are associated with CVD [32,33] due to their role in atherosclerosis formation and progression [34]. In addition, increasing data suggest that fructose can alter lipid metabolism genes expression, including those genes that reduce the removal of hepatic fat and increase its accumulation, highlighting an epigenetic effect of fructose, particularly on NAFLD [35].

**Table 1:** Preclinical and clinical studies evaluating fructose consumption on cardiometabolic parameters.

Study	Year	Primary Endpoint	Main Findings
Teff KL et al. [8] Dietary fructose decreases the circulating insulin and leptin, attenuates postprandial suppression of ghrelin, and increases triglycerides in women	2004	Testing whether meals high in fructose (HFr) would result in lower leptin concentrations than meals containing the same amount of glucose (HGl)	Consuming HFr beverages with meals results in lower circulating insulin and leptin concentrations and higher ghrelin and triglyceride levels compared with consumption of HGl beverages
Aeberli I et al. [29] Fructose intake is a predictor of LDL particle size in overweight schoolchildren	2007	To determine whether LDL particle size is associated with fructose intake in normal-weight and overweight children	Greater total and central adiposity are associated with smaller LDL size and lower HDL cholesterol. Additionally, higher fructose intake predicts smaller LDL particle size
Stanhope KL et al. [25] Consuming fructose-sweetened, not glucose-sweetened, beverages increases visceral adiposity and lipids and decreases insulin sensitivity in overweight/obese humans	2009	To assess the relative effects of fructose-sweetened and glucose-sweetened beverages on lipid and glucose metabolism in humans, overweight, and obese subjects	Dietary fructose increases hepatic de novo lipogenesis, promotes dyslipidemia, decreases insulin sensitivity, and increases visceral adiposity in overweight/obese adults
Cox CL et al. [26] Consumption of fructose- but not glucose-sweetened beverages for 10 weeks increases circulating concentrations of uric acid, retinol-binding protein-4, and gamma-glutamyl transferase activity in overweight/obese humans	2012	Investigating the relative effects of 10 weeks of fructose or glucose consumption on plasma uric acid, retinol binding protein-4, and liver enzyme activities in men and women	Consumption of fructose, but not glucose, led to significant increases in 24-h uric acid profiles and retinol binding protein-4 concentrations, as well as plasma gamma-glutamyl transferase activity
de Castro UG et al. [11] Age-dependent effect of high-fructose and high-fat diets on lipid metabolism and lipid accumulation in liver and kidney of rats	2013	Evaluating biochemical, physiological, histological, and biometric parameters in rats with a high-fat or high-fructose diet	High-fructose diet caused the most significant change in the metabolism of serum lipids and lipid accumulation in the liver and kidney, while a high-fat diet induced elevation of arterial pressure and heart rate and increased visceral lipid stores
Morenga LA et al. [24] Dietary sugars and cardiometabolic risk: systematic review and meta-analyses of randomized controlled trials of the effects on blood pressure and lipids	2014	Systematic review and meta-analysis of randomized controlled trials that examined effects of the modification of dietary free sugars (mostly fructose) on blood pressure and lipids	Higher intake of sugars is associated with increased concentrations of triglycerides, total and LDL cholesterol, and blood pressure (this last effect was significant in studies of a longer duration only)
Yoo S et al. [21] High dietary fructose intake on cardiovascular disease-related parameters in growing rats	2016	Determining the effects of a high-fructose diet on cardiovascular disease-related parameters in growing rats	High-fructose diet increased total-fat weight and serum triglyceride levels. Negative effects were found in abdominal aortic thickness, as well as endothelial nitric oxide synthase and endothelin-1 mRNA expression
Gungor A et al. [31] The relationship between daily fructose consumption and oxidized low-density lipoprotein and low-density lipoprotein particle size in children with obesity	2021	To investigate the relationship between fructose consumption and obesity and the role of fructose consumption in development of atherosclerosis in obese and healthy children	The overconsumption of fructose in children triggers atherogenic diseases by increasing the levels of small, dense LDL

## 4. Conclusions

Although it is known that increased consumption of added sugars is associated with greater risk of CVD independent of caloric intake, sugar consumption remains high worldwide. Since high fructose intake is associated with several cardiometabolic alterations, including dyslipidemia, insulin resistance, diabetes, hyperuricemia, and NAFLD, further longitudinal studies should be conducted in order to fully elucidate the association between certain sugars, like fructose, and CVD development and progression. This has a particular relevance during the current coronavirus pandemic since an increased prevalence of cardiometabolic diseases and altered lifestyle has been observed, including altered nutritional habits due to repeated lockdowns, increased teleworking, and significant changes in the socioeconomic conditions worldwide [36,37]. It is therefore proper time to improve nutritional habits, reducing the overconsuptions of sugars that may be deleterious for cardiometabolic diseases, such as fructose.
